# Hospital Notes

**Published:** 1894-06

**Authors:** 


### Hospital Notes.

---

AT THE Fitch Hospital, Edward R. Haydenbrook, M. D., has been appointed senior house surgeon, and John Chalmers, M. D., has been appointed junior house surgeon; J. A. Jackson and George J. Hallen have been appointed ambulance surgeons to November 1, 1894, and R. V. Crittenden has been appointed substitute; W. G. Russell and U. B. Stein have been appointed ambulance surgeons, November 1, 1894, to May 1, 1895, with W. C. Keys as substitute.

---

AT THE Sisters of Charity Hospital, Drs. Max Keiser and Jacob R. Baxter passed highest in the competitive examination for house physicians, and have been appointed as such. These two men are graduates of Niagara Medical College. Dr. Baxter is the only son of Dr. Jacob Baxter, speaker of the Ontario House of Commons.

---

AT THE Buffalo General Hospital the following appointments have been made: John Riordan, M. D., house surgeon, promoted from junior assistant; Ernest L. Ruffner, M. D., junior house surgeon; Chauncy Lamb, M. D., house physician, promoted from junior assistant.

